# Meta-synthesis and science mapping analysis of HIV/HPV co-infection: a global perspective with emphasis on Africa

**DOI:** 10.1186/s12992-022-00812-w

**Published:** 2022-03-24

**Authors:** Hope Onohuean, Eric O. Aigbogun, Bright E. Igere

**Affiliations:** 1 Biopharmaceutics Unit, Pharmacology and Toxicology, School of Pharmacy, Kampala International University, Western Campus, Ishaka-Bushenyi, Uganda; 2 Biomolecules, Metagenomics, Endocrine and Tropical Disease Research Group (BMETDREG), Kampala International University, Western Campus, Ishaka-Bushenyi, Uganda; 3 Department of Human Anatomy, Faculty of Biomedical Sciences, Kampala International University, Western Campus, Ishaka-Bushenyi, Uganda; 4 Department of Microbiology and Biotechnology, Western Delta University Oghara, Oghara, Delta State Nigeria

**Keywords:** HPV/HIV co-infection, Public health concern, meta-synthesis, Mapping analysis, Africa, Research advancement

## Abstract

**Background:**

Viral infections are emerging with diverse clinical relevance both in endemic environments and non-endemic regions of the world. Some of the viruses cause co-infections that are of public health importance. The progress of studies on human immunodeficiency virus / Human papillomavirus (HIV/HPV) co-infection is not well documented especially in Africa where cases are endemic.

**Method:**

Using Preferred Reporting Items for Systematic Reviews and Meta-Analyses (PRISMA) guidelines, we conducted a global three-decade meta-synthesis and science mapping analysis on HIV/HPV co-infections. Assessment of progress, Author/Country productivity/trends, topic conceptual framework, and international collaborative networks were analyzed.

**Results:**

We recovered 196 documents of 115 sources from the web of science database. The meta-synthesis revealed 1203 prolific authors containing nine solo authors, an annual growth rate of 8.09%, a significant average citation per article of 20.7%, and an average citation per year per document of 2.1. A significant high correlation between the mean/TC per article and the mean total citation (TC) per year showed 80.98% of the articles produced between 2005 and 2007 on HPV/HIV co-infection. The co-author per document index were 7.0 and the collaboration index was 6.4. The meta-analysis also revealed inadequate funding from individual or governmental organizations; among the 196 documents dataset, 114 (58.2%) were funded, and only 31 (15.8%) were funded in Africa where HIV/HPV co-infection cases are endemic.

**Conclusions:**

Authors’ collaboration network, countries’ collaboration, authors’ citations and implementation of research-based finding in previous studies are yet to receive the relevant outcome, especially as various countries in the African continent have received poor funding with a repeated reporting of co-infection associated with HIV/HPV. African needs to re-awaken and stir up research-based interest in HPV/HIV co-infection studies to resolve indigenous public health concerns associated with the viral endemicity.

## Background

As of 2018, World Health Organization (WHO) describes the human immunodeficiency virus (HIV) as a ‘global epidemic’ due to the increasing distribution of the infection with types 1 [24, 133], and HIV-2 has had a limited worldwide extension and is primarily confined to West Africa, infecting an estimated 1–2 million people [45], similar to other Africa endemic public health concern such as *Vibrio* Cholera [95], Malaria [93], Drug resistance [94]. Meanwhile, HIV (type 1 & 11) epidemic has affected about 76 million people with recorded death being more than 33 million people at the end of 2019 [120]. Currently, 31.6–44.5 million people are living with HIV globally [132, 133]. An estimated 0.7% [0.6-0.9%] of adults aged 15–49 years worldwide are infected with HIV, although the burden of the epidemic continues to vary considerably between countries and regions [24]. According to WHO (2018), African region remains most severely affected, with nearly 1 in every 25 adults (3.7%) [131] living with HIV, which accounts for more than two-thirds of the people infected with HIV worldwide [108]. More challenging and of greater public health threat is the coinfection/comorbidity recorded for HIV and Human papillomavirus (HPV) infections. Human papillomavirus (HPV) has been reported as one major co-infecting virus with HIV infections in various regions of Africa and other continents where it is endemic [11, 16, 124, 130]. It is responsible for several diseases ranging from common benign warts to invasive carcinoma at various anatomical sites of infection, including the cervix, vulva, vagina, penis, anus, and oropharynx [11, 16, 124, 130] which are communally distributed sexually. Both HPV and HIV type 1 infections have been classified as carcinogens [47, 130], by the International Agency for Research on Cancer (IARC). The Wheeler reports have shown that HPV is a direct carcinogen whereas HIV-1 is an indirect carcinogen via immune suppression [130]. Similarly, other investigators’ [42, 97, 105] reports have revealed that HPV infection is the etiological agent responsible for general cervical cancers including a subset of cancers of the anus, oropharynx, penis, vagina, and vulva. In addition, individuals with HIV and acquired immunodeficiency syndrome (AIDS) are at a high risk of developing HPV related cancers [5, 23, 38, 96, 135].

In recent times, reports from various investigations have shown HIV/HPV co-infection of both viruses in clinical cases. HIV/HPV co-infection is shown to be ravaging lives of many living in resources limited settlements, and conducting integrated analyses [12, 110], of global scientific relevance is useful in gaining more insight into the current position and emerging trends [136].

In the USA, reports have revealed that there is a high incidence of cancer-related infections among HIV- infected patients with a simultaneous post-report of HPV infection amongst the general population [34, 115]. In South Africa, HPV/HIV-infected individuals have 29 times risk of developing anal cancer, about 6 times chances for vulvar/vaginal cancer including cervical cancer, and 4 times chances of developing penile cancer [60]. In Italy, prevalence of HIV/HPV co-infection was 8.8% [101] to 38% base on the population of the area examined for HPV/HIV coinfections [8]. However, in sub-Sahara African there is a gap in literature on the potential clinical cases of HPV-related cancers among HIV-infected patients. Although the core of HIV-infected women were documents from sub-Sahara African region with cervical cytological of infected HPV, which is estimated to be one in every five women [15].

Recently, there has been a decrease in the HIV morbidity and mortality since the introduction of antiretroviral therapy (ART) and highly active antiretroviral therapy (HAART) [53, 85] for the management of early HIV infection cases. Whereas many HIV-associated comorbidities and HPV-related diseases continue to rise with higher mortality in low and middle-income countries, the impact of ART and HAART is seldom observed amongst HPV/HIV related cases. Many studies have shown controversies in the use of highly active antiretroviral therapy (HAART) for the treatment of HIV/HPV coinfection, neoplasia, and have indicate HPV-associated infection HAART [49, 98]. In fact no study has indicate positive reductive effect of HAART in the progression of high-grade cervical intraepithelial neoplasia (CIN) or anal intraepithelial neoplasia (AIN) from HPV/HIV coinfected individuals [30, 49, 60]. To the best of our knowledge, no study has examined HIV/HPV coinfection and related dysplasia in a global or regional context, and HAART effectiveness in its management using integrated content analytics and science mapping bibliometrics analytics. In the same vain, there is very scanty information on strategies of mitigating the public burden of HIV/HPV co-infection, in addition to unknown assessment of literature that contributes to this subject. Therefore, we investigated the breadth and progression of research knowledge on HIV/HPV coinfection to stir up researchers, policy makers and funders to key into research strides on reducing HIV/HPV co-infection as well as other associated clinical cases. The study also use literature to support the explanation and interpretation of HIV/HPV world problem, with emphasis on research progress and sustenance funds / grants in one of the endemic region Africa.

The science mapping hinged on Author/country, productivity/trends, topic conceptual framework, and international collaborative networks were analyzed. Integrated content analytics and science mapping bibliometrics analysis remains a useful research tool with an increasing potential for monitoring, handling, supporting and appraising decisions in scientific/technological progress and research development [99, 110]. In addition, diverse investigators have applied bibliometric media as evidence base strategy for redirecting research focus [27, 28, 44, 64, 70, 84, 92]. In this regard, we use integrated content analytics and science mapping analytics to map scientific progress related to HIV/HPV coinfection as an ancillary source-track of epidemic spanning the period of 1990 to 2019. In addition, the study also identified authors production/production trends, inter institutional/international research activities, conceptual topic thematic and gaps for future prospects.

## Data and method

### Data collection

The “Preferred Reporting Items for Systematic Reviews and Meta-Analyses” (PRISMA) guidelines were used to search the Web of Science (WoS) database for relevant studies on HIV/HPV/Coinfection [80].

**Fig. 1:**
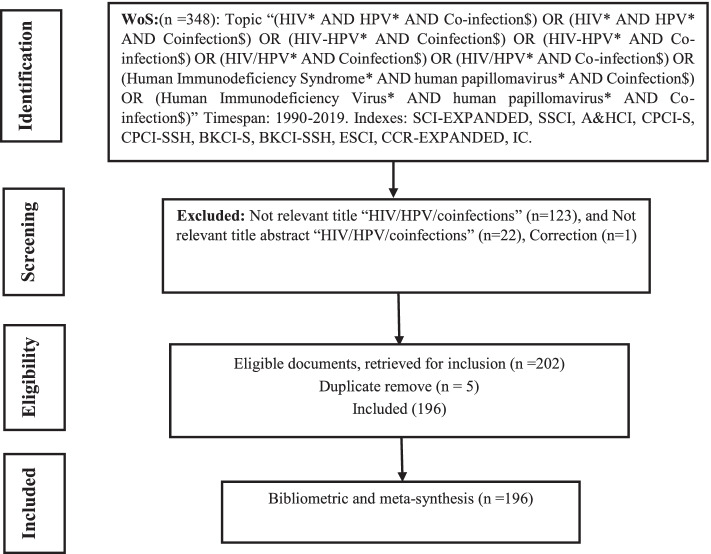
PRISMA process of searching, reviewing and meta-synthesis of articles on HIV/HPV/Coinfection research domains

We collated published articles, editorial material, meeting abstract, proceedings paper, review article, on HIV/HPV co-infections from global repository data by searching the Web of Science (WoS) core collection database on 19th July 2020 at about 10.25 GMT + 2). The Boolean topic search approach applied included “(HIV* AND HPV* AND Co-infection$) OR (HIV* AND HPV* AND Coinfection$) OR (HIV-HPV* AND Coinfection$) OR (HIV-HPV* AND Co-infection$) OR (HIV/HPV* AND Coinfection$) OR (HIV/HPV* AND Co-infection$) OR (Human Immunodeficiency Syndrome* AND human papillomavirus* AND Coinfection$) OR (Human Immunodeficiency virus* AND human papillomavirus* AND Coinfection$) to recoup all available documents on the subject, “HIV/HPV co-infection” between 1990 and 2019. The Web of Science Core Collection database adopted in this study has a high reputable academic research database covering thousands of journals, books, conferences as well as millions of records from clarivate.libguides.com [2, 50, 62, 129, 137]. To ensure the inclusion of abbreviated or shorten words, such as “HIV”, “HPV”, “AIDs”, and any other related words the wildcard * and $ where added to the end of the search algorithms. Thereafter, all document that meet the eligibility criteria for HIV/HPV co-infections were extracted in BibTex file format and the authors, titles, abstracts mined in PDF file format. Only the articles that have the search term in their title and abstract were qualified for inclusion detailed in Fig. 1.

### Data analytics

All the bibliometric variables were retrieved filtered and normalized for quality control. The results were analyzed in biblioshiny package [6, 51, 127] on Rstudio versions 3.5.1 [103] and Microsoft excel 16, while the network maps were visualized in VOSviewer software1.6.13 [125].

## Results

Our study recovered a total of 196 documents relevant to HIV/HPV co-infection from the web of science database within the survey period (1990 – 2019), with informatics and attributes as presented in Table 1. A total of 1203 prolific authors distributed as 1194 multi-authored documents and nine solo authors were involved in the retrieved studies. Other informatics of published documents include: 2 (1%) editorial material, 5 (2.5%) meeting abstract, 5 (2.5%) proceedings paper and 21 (10.7) reviews. We were not surprised of finding no new documented items captured in newsletters, since HIV/HPV co-infection is not an outbreak infection/disease. The average citation per document is 20.7, with 0.2 document/author (6.1 authors/document), 7.0 co-authors/document, and a collaboration index of 6.4 which implies a high participation of co-authorship per document [107]. Authors who published alone in related studies were 9, whereas 1197 authors publications were shown as multi-authored documents (Table 1). The funding and supporting organisations are highlighted in Table 2. Among the 196 documents that met the criteria for assessment in this study, only 114 of the study received funding support from public/governmetal and private organisations of which 31 (15.8%) studies conducted and funded in Africa. This indicate a reduced funding interest in the region despite the emdemic nature of the virus.

**Table 1:** Data Summary and key information from Web of Science database on HIV/HPV co-infection publications

Description	Count/Rate		Count/Rate
Main data information		Document type	
Timespan	1990:2019	Article	163
Sources (Journals, Books, etc)	115	Editorial material	2
Documents	196	Meeting abstract	5
Average years from publication	8.1	Proceedings paper	5
Average citations per documents	20.7	Review	21
Average citations per year per doc	2.1		
References	6246		
		Keywords Plus (ID)	679
**Author Information**		Author’s Keywords (DE)	418
Authors	1203		
Author Appearances	1372		
Authors of single-authored documents	9		
Authors of multi-authored documents	1194		
Documents per Author	0.2		
Authors per Document	6.1		
Co-Authors per Documents	7.0		
Collaboration Index	6.4

**Table 2:** Africa funder and support organisations of HIV/HPV co-infection studies from 1990 to 2019

Authors	DOI	Funders and Grant Number	Publication Source	Region/Continent/Countries
Chambuso et al., 2019 [19]	10.3389/fonc.2019.00951	National research foundation of South Africa (NRF) (grant number: 64815)	Frontiers in Oncology	South Africa
Nelson et al., 2019 [86]	10.1186/s12879-019-3925-3	Canadian Institute Of Health Research Grant \#Het-85,518,Ontario Hiv Treatment Network Grant \#Ahrc G-1066 And Ontario Hiv Treatment Network Knowledge, Translation And Exchange Grant, University Of Rochester Center For Aids Research, An Nih-Funded Program (P30 Ai078498).	BMC Infectious Diseases	African-Caribbean
Marembo et al., 2019 [69]	10.1159/000502206	National Aids Council And The Department Of Medical Microbiology (Uz)	Intervirology	Zimbabwe
Chambuso et al., 2019 [19]	10.7150/jca.25600	Postgraduate Academic Mobility For African Physician-Scientists (Pamaps), European Union.; South African Medical Research Council, Clinical Gynaecological Cancer Research Centre, University Of Cape Town, South Africa.; National Research Foundation Of South Africa (Grant Number: 64815); National Health Laboratory Service Of South Africa (Grant Number: 004\_94678)	Journal of Cancer	South Africa
Campos et al., 2018 [17]	10.1097/QAI.0000000000001778	National Cancer Institute (U01ca199334) And The Bill \& Melinda Gates Foundation (Opp1086544). United States Agency For International Development (Usaid), Award Number Aid-674-A-12-00029;	Jaids-Journal Of Acquired Immune Deficiency Syndromes	South Africa
Mandishora et al., 2018 [67]	10.1016/j.pvr.2018.04.006	Letten Foundation Grant	Papillomavirus Research	Harare, Zimbabwe
Menon et al., 2018 [77]	10.1186/s12985-018-0961-3	Fonds Wetenschappelijk Onderzoek (P175/10/2005). The Fp7-Funded Research Consortium (Hpv-Ahead) And The Interreg-Supported Elektron Consortium	Virology Journal	Mombasa, Kenya
Menezes et al., 2018 [76]	10.1136/sextrans-2016-053046	Merck Sharp \& Dohme. Merck (Iisp39582)	Sexually Transmitted Infections	Western Cape, South Africa
Mandishora et al., 2017 [66]	10.1002/jmv.24825	Letten Foundation Norway	Journal of Medical Virology	Harare, Zimbabwe
Meiring et al., 2017 [74]	10.1016/j.pvr.2017.05.001	Poliomyelitis Research Foundation [Grant Number 11/24] And South African Research Chairs Initiative of The Department Of Science And Technology [Grant Number 64815]. Tlm Was Supported By Awards From The National Research Foundation South Africa And Clinical Infectious Diseases Research Initiative (Wellcome Trust).	Papillomavirus Research	South Africa
Ginindza et al., 2017 [43]	10.1371/journal.pone.0170189	University Of Kwazulu-Natal College Of Health Sciences Doctoral Research, And Health And Welfare Sector Education And Training Authority (Hwaseta). Open Access Publication From The Victor Daitz Information Gateway, An Initiative Of The Victor Daitz Foundation And The University Of Kwazulu-Natal.	Plos One	Swaziland.
Menon et al., 2017 [78]	10.1186/s13027-016-0114-5	The Vlir Iuc Moi University	Infectious Agents And Cancer	Kenya
Ermel et al., 2016 [35]	10.1186/s13027-016-0102-9	HPV Signature Center, Indiana University School of Medicine (D. Brown).	Infectious Agents And Cancer	United States, Kenya, or Botswana.
He et al., 2016 [48]	10.3390/v8090245	By Charitable Donations From The Humane Research Trust, The Janice Cholerton Post Graduate Support Fund/East Africa Medical Trust (Eamt), The Caring Cancer Trust, And The Cancer Prevention Research Trust.	Viruses-Basel	Sub-Sahara African
Mueller et al., 2016 [82]	10.1186/s12879-016-1706-9	The Global Disease Detection (Gdd) Programme Grant No. 1u19gh000622-01 While The Main Study Received Funding and Support From Pepfar/Usaid And The Anova Health Institute Npc.	BMC Infectious Diseases	Cape Town, South Africa
Kriek et al., 2016 [55]	10.1016/j.virol.2016.03.022	The Cancer Association of South Africa (Cansa) [Cancer Research Grant: 2011 Jsp]. The Fogarty Foundation (Shape Programme) and The South African Medical Research Council (Mrc) National Health Scholars Programme	Virology	Gugulethu, Cape Town, South Africa
Adler et al., 2016 [1]	10.1155/2016/7310894	The National Institute of Allergy and Infectious Diseases at The National Institutes of Health (5 K23ai07759 To David Adler).	Advances In Virology	Gugulethu, Cape Town, South Africa
Belglaiaa et al., 2015 [9]	10.1186/s13027-015-0040-y	The University of Franche-Comte, Ligue Contre Le Cancer (Ccir-Ge) and Doctoral School ``Environnements-Sante” Besancon, France	Infectious Agents And Cancer	Souss area, Morocco.
Sobota et al., 2014 [109]	10.1186/1750-9378-9-26	Public Health Service Award T32 Gm07347 From The National Institute of General Medical Studies For The Vanderbilt Medical-Scientist Training Program	Infectious Agents And Cancer	Botswana
Ameur et al., 2014 [4]	10.1038/srep04398	The Swedish Research Council, The Swedish Cancer Foundation, The Swedish International Agency For Development and The Knut And Alice Wallenberg Foundation, Based on Research Supported By The South African Research Chairs Initiative of The Department Of Science And Technology and National Research Foundation	Scientific Reports	South Africa
Rositch et al. 2014 [102]	10.1097/QAD.0000000000000092	The National Cancer Institute, National Institutes of Health (Grant R01 Ca114773-04) and The Unc Center For Aids Research (Grant P30 Ai050410 From The National Institute of Allergy and Infectious Diseases, National Institutes of Health), The Division of Aids, National Institute of Allergy and Infectious Diseases, National Institutes of Health (Grant Ai50440), The Canadian Institutes of Health Research, and The Chicago Developmental Center For Aids Research (D-Cfar), an NIH Funded Program (P30 Ai 082151), an NIH Postdoctoral Training Grant in Cancer Epidemiology (Grant T32 Ca009314).	AIDS	Kisumu, Kenya
Mbulawa et al., 2014 [72]	10.1186/1471-2334-14-51	Poliomyelitis Research Fund, Medical Research Council, Swedish International Development Cooperation Agency, Swedish Cancer Foundation, Cancer Association of South Africa, National Health Laboratory Services, National Research Foundation And South African Research Chairs	BMC Infectious Diseases	Empilisweni centre, Gugulethu, Cape Town, South Africa
Vaccarella et al., 2013 [122]	10.1186/1750-9378-8-50	The Washington Global Health Alliance, The National Institutes of Health (Grant Number 5k23ai065222-04), A Grant From The Bill \& Melinda Gates Foundation (Grant Number 35537), and From The Fondation De France (Grant Number 00016673).	Infectious Agents And Cancer	Kenya
Sudenga et al., 2013 [117]	10.1016/j.humimm.2013.08.010	National Institutes of Health Cancer Prevention and Control Training Grant (S.L.S. R25ca47888). Research Funded by U01-Hd32830, 5p30 Ai27767-20, Uab CFAR/CCC Malignancy Pilot Project Award	Human Immunology	African-American
AKAROLO-ANTHONY	10.1186/1471-2334-13-521	The Um-Capacity Development For Research in Aids Associated Malignancy Grant (NIH/NCI 1d43ca153792-01) To CAA. Hpv Genotyping Was Supported By Niaid U19 Ai084081 To Cmw	Infectious Diseases	Abuja, Nigeria
SAHASRABUDDHE et al., 2012 [104]	10.1158/1940-6207.CAPR-11-0540	Cancer Prevention Research	Cancer Prevention Research	Lusaka, Zambia
VELDHUIJZEN et al., 2011 [128]	10.1186/1471-2334-11-333	The European and Developing Countries Clinical Trials Partnership (EDCTP)	BMC Infectious Diseases	Kigali, Rwanda
RAMOGOLA-MASIRE et al., 2011 [100]	10.1097/PGP.0b013e31821bf2a6	The NIH CFAR Grant NIH Ip30 AI 45008 and Health Services and Human Research Grant 1u2gps001949 (President’s Emergency Plan For Aids Relief).	International Journal Of Gynecological Pathology	Botswana
MACLEOD et al., 2011 [65]	10.1002/jmv.22178	Eunice Kennedy Shriver National Institute of Child Health \& Human Development (To C. Wester); Grant Number: K23hd049292	Journal Of Medical Virology	Botswana
Tobian and Gray, 2011 [121]	10.2217/FMB.11.59	The Johns Hopkins University Clinician Scientist Award.	Future Microbiology	Sub-Sahara African
Mueller et al., 2010 [81]	10.1136/sti.2009.037598	The National Institute For Communicable Diseases, National Health Laboratory Service, South Africa.	Sexually Transmitted Infections	Johannesburg, South Africa

**Fig. 2:**
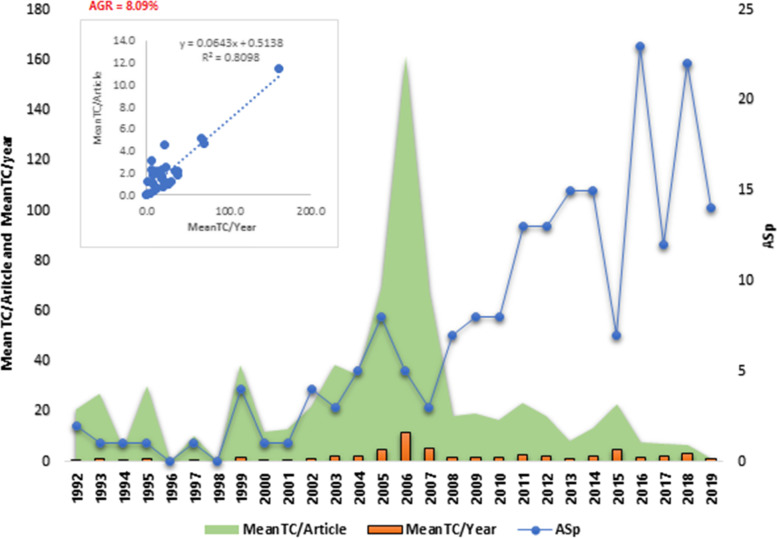
Yearly distribution of scientific productions and citations. MeanTC/Article = Mean Total Citation per articles; MeanTC/year = Mean Total citations per year; AS_p_ = Annual Scientific Publications; AGR = Annual Growth Rate. Source: *Biblioshiny* compilation form Web of Science database (correlation calculations in excel by Author)

From Fig. 2, The annual growth rate was 8.09%, and a significant observation was the corresponding peak and fall in the MeanTC per article, meanTC per year and annual scientific production between 2005 and 2019. The significant high correlation between the mean/TC per article and the mean total citation (TC) per year showed that 80.98% of the articles on HPV/HIV co-infection have remained scientifically relevant, although the interest has been dropping in the third decade (with regard to meanTC per article) when compared to the second decade. This indicates that the article produced between 2005 and 2007 were much more scientifically relevant to the subject matter with appropriate attention than any other year and thus gained more interest from the scientific community.

The Table 3 reveals leading publication journals on studies related to HIV/HPV co-infection amongst other published documents. The PLOS one ranked top based on the TP; however, observing the global total citation (TC) and total local citation (TLC), Sexually Transmitted Infections journal maintained fourth (4th) rank while Journal of infectious disease ranked first. The PLOS one was top ranked for TC but fell to 8th rank for TLC, Clinical Infectious Diseases journal had the highest score of 70.8 (approximately 71 for each of its publication) for the calculated average local citation per article (aC/p). In addition, a very close value for the total local citation per publication (TLC/p) was observed, nevertheless, LANCET and PLOS ONE had the highest point of 40.5 and 40.0 respectively.

**Table 3:** Top 10 Leading Journals in HIV-HPV co-infection research based on 20 searched sources

Ranked by TP					Ranked by TLC			
Rank	Journal (PY)	TP	TC	aC/p	Rank	Journal	TLC_**s**_	TLC/p
1	Plos One (2010)	17	251	14.8	1	J Infect Dis	417	24.5
2	BMC Infect Dis (2009)	10	137	13.7	2	Aids	328	32.8
3	Infect Agents Cancer (2013)	7	59	8.4	3	Int J Cancer	237	33.9
4	Sex Transm Infect (2009)	6	94	15.7	4	Sex Transm Infect	171	28.5
5	J Med Virol (2004)	5	100	20.0	5	New Engl J Med	167	33.4
6	Clin Infect Dis (1995)	4	283	70.8	6	Lancet	162	40.5
6	Gynecol Oncol (2010)	4	225	56.3	7	Plos One	160	40.0
6	Int J Std Aids (2006)	4	40	10.0	8	J Virol	144	36.0
6	Memorias I O Cruz (2005)	4	52	13.0	9	J Clin Microbiol	130	32.5
6	Papillomavirus Res (2017)	4	16	4.0	10	Clin Infect Dis	122	30.5

*TP* total publication, *TC* total citation, *aC/p* Average Citation per Publication, *TLC/p* Average Total Local Citation per Publication

*BMC INFECT DIS* BMC Infectious Diseases, *INFECT AGENTS CANCER* Infectious Agents and Cancer, *SEX TRANSM INFECT* Sexually Transmitted Infections, *J MED VIROL* Journal of Medical Virology, *CLIN INFECT DIS* Clinical Infectious Diseases, *GYNECOL ONCOL* Gynecologic Oncology, *INT J STD AIDS* International Journal of STD & AIDS, *MEMORIAS I O CRUZ* Memórias do Instituto Oswaldo Cruz, *PAPILLOMAVIRUS RES* Papillomavirus Research, *J INFECT DIS* The Journal of Infectious Diseases, *INT J CANCER* International Journal of Cancer, *J VIROL* Journal of Virology, *J CLIN MICROBIOL* Journal of Clinical Microbiology

In Table 4, the top 15 leading authors, their research impact and turnout are described from the viewpoint of local citation. Nicol et al. [87], have a 14 years publishing timeline just as Minkoff (2005) and Grinsztejn B [46]; however, more publications had made his local presence recognised, but in terms of contribution turnout, Minkoff’s research seems to be more relevant (TC = 365) than any other researcher (Nicol and Grinsztejn) on HIV/HPV co-infection despite having 3 publications. This may be attributed to the quality of the published document, the global impact, local, community and related research relevance.

**Table 4:** Top 15 leading authors research impact and contribution turnout from the local presence

S/N	Author (Y-Start)	PTL	TC	NP	TC/p	TC/P/t
**1**	Nicol AF (2005)	14	127	12	10.58	0.76
**2**	Williamson AL (2002)	17	103	8	12.88	0.76
**3**	Grinsztejn B (2005)	14	105	7	15.00	1.07
**4**	Nuovo GJ (2006)	13	63	7	9.00	0.69
**5**	Russomano F (2006)	13	80	6	13.33	1.03
**6**	Fernandes ATG (2002)	17	62	5	12.40	0.73
**7**	Menon S (2016)	3	22	5	4.40	1.47
**8**	Tristao A (2002)	17	65	5	13.00	0.76
**9**	Friedman RK (2006)	13	55	4	13.75	1.06
**10**	Mbulawa ZZA (2009)	10	84	4	21.00	2.10
**11**	Rossi R (2016)	3	21	4	5.25	1.75
**12**	Denny L (2016)	3	19	3	6.33	2.11
**13**	Mabeya H (2016)	3	20	3	6.67	2.22
**14**	Meiring TL (2014)	5	30	3	10.00	2.00
**15**	Minkoff H (2005)	14	365	3	121.67	8.69

*PTL* Production timeline, *Y-Start* Year started publishing, *TC/P* Average citation, *TC/P/t* Average citation-timeline ratio (contribution turnout)

Source: Biblioshiny compilation form Web of Science database, Authors computation (in blue)

In Table 5, the top 15 authors (as per a single relevant article or publication), their scientific global presence, and performance indices were presented. Munoz [83], lead in both the TG indices (58.5) and the performance citation index. In this article, we measured the global to local citation as a way of indexing the global to local citation and this showed that there is more global than local interest in HIV/HPV related researches. Munoz [83], presence is approximately two and a half times greater in TGC per year and almost ten times greater in G/LCi than the next on the range order as well as any other author in this research area. We clustered and visualized the network of authors keywords to identified sub-fields which represent sub-set base on concept-similarity profile [126]. However, many publications may be found in more than one sub-field, this overlap between sub-fields by joint of publications are used to calculate a further co-occurrence matrix, on the basis of the identified subfield publication similarity. Map networks are construct and clusters by multidimensional scaling, whereby high similarity are placed in each other’s domain’s, while sub-fields of low similarity are distant from each other. The size of a sub-field indicates the volume of publications in relation to the subject. Furthermore, the strength of the relationship between two individual subfields are indicated by a connecting line.

**Table 5:** Top 15 authors with significant global presence, production turnout, and performance indices

Performance - TGC^**(a,b)**^	TGC	TGC/Year	GC/t	Performance- CI^**(a,b)**^	LC	GC	G/LCi
Munoz N (2006) [83]	760	50.67	58.5	Munoz N (2006) [83]	5	760	152.0
Strickler HD (2005) [116]	319	19.94	22.8	Vajdic CM (2009) [123]	3	59	19.7
Steben M (2007) [114]	160	11.43	13.3	Ferenczy A (2003) [36]	4	75	18.8
Silverberg MJ (2015) [111]	127	21.17	31.8	Sobhani I, 2004) [112]	4	59	14.8
Bessesen M (1999) [10]	123	5.59	6.2	Mbulawa ZZA (2009) [71]	6	51	8.5
De Pokomandy A (2011) [32]	115	11.50	14.4	Veldhuijzen NJ (2011) [128]	4	32	8.0
Ferenczy A (2003) [36]	75	4.17	4.7	Gao L (2010) [40]	6	47	7.8
Almonte M (2008) [3]	74	5.69	6.7	Mendez-Martinez R (2014) [75]	4	28	7.0
Chaturvedi AK (2005) [20]	70	4.38	5.0	Macleod IJ (2011) [65]	4	20	5.0
Clarke B (2002) [21]	65	3.42	3.8	Garbuglia AR (2012) [41]	6	25	4.2
Baay MFD (2004) [7]	60	3.53	4.0	Nicol AF (2005) [88]	8	28	3.5
Vajdic CM (2009) [123]	59	4.92	5.9	Nicol AF (2006) [90]	3	10	3.3
Sobhani I (2004) [112]	59	3.47	3.9	Lee BN (1999) [59]	4	13	3.3
Kwong Jc (2012) [56]	57	6.33	8.1	Nicol AF (2008) [89]	5	15	3.0
Dos Ramos Farias MS (2011) [33]	54	5.40	6.8	Nicol AF (2008) [89]	9	18	2.0

^a,b^ Name, Publication year; *TGC* Total Global Citation, *GC/t* Global Citation turnout, *LC* Local Citation, *GC: G/LCr* Global-Local citation index (CI)

Source: Biblioshiny compilation form Web of Science database, Authors computation (in blue)

### Conceptual factorial analysis (relatedness of research terminologies)

**Fig. 3:**
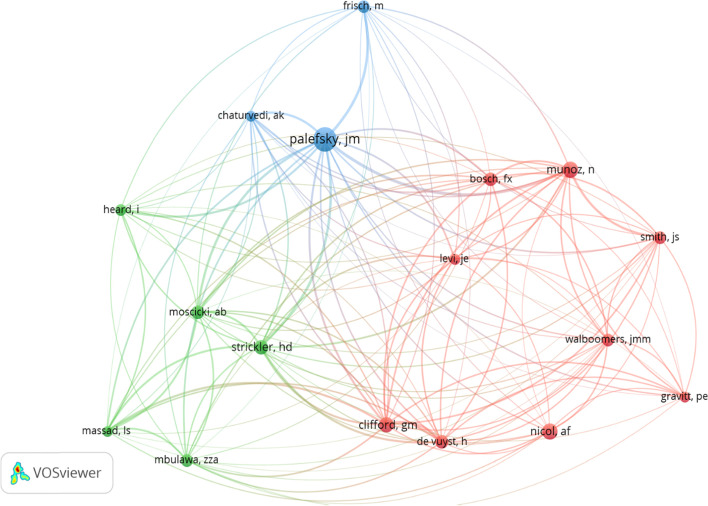
Authors co-citation network Cluster 1 (9) [red]; Cluster 2 (5) [green], Cluster 3 (3) [blue]). Note: The minimum author’s citation threshold was set at 20 and of the 4687 authors, only 17 met the criteria. The bubble size indicates the most cited author, while the colour refers to the clustering and line thickness the strength of the corresponding link

Author co-citation network map is shown in Fig. 3. Palefsky JM is the most significant author that is linked to other authors. This indicates that in related studies, such author associates earlier report and relates them to previous similar studies hence a significant association of co-authors citation. The collaboration link of Palefsky JM., was rather weaker than that of Munoz N., which had lots of collaborating links within and outside the cluster.

This network confirms the position of the Authors as regards HIV/HPV coinfection research; that Munoz’s global visibility and relevance in HIV/HVP co-infection studies are attributed to researchers being specific about the content of the research than the number of publications from the research.

**Fig. 4:**
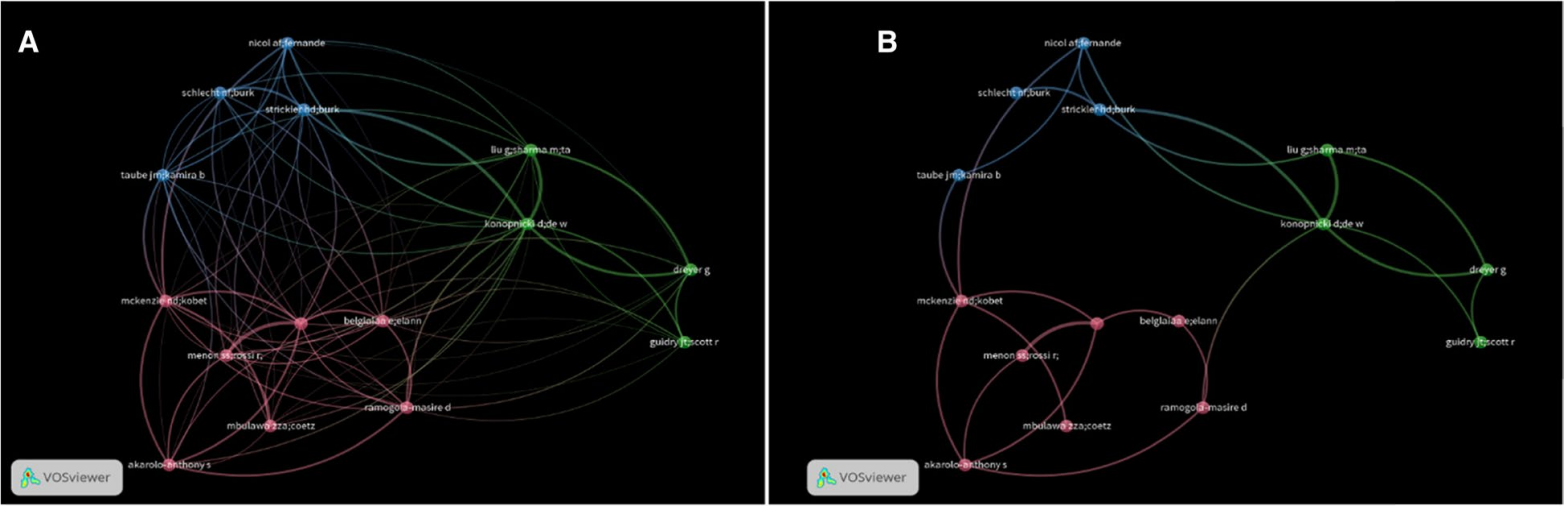
Authors collaboration network (**A**) Minimum of 1 (**B**) minimum of 5 (cluster 1: 7, Cluster 2: 4, and Cluster 3: 4). Note: The minimum author’s collaboration threshold was set at 1, then 5, and out the 4687 authors, only 15 met the criteria. The bubble size indicates the most cited author, while the colour refers to the clustering and line thickness the strength of the corresponding link (There is an unlabelled author and this was marked as Anonymous in biblioshiny). Source: VOSViewer compilation form Web of Science database

In Fig. 4A, the largest cluster is 7, while the biggest network is cluster 2, which account for the largest author’s collaboration. At minimum threshold of 1 collaboration, only 15 authors networked among themselves. However, when the threshold is raised to a minimum of 5 collaborations, the links are scanty and it can be observed that just a single author links the entire cluster (Fig. 4B). Despite the amount of grant that has been awarded for HIV/HPV co-infection research and the numerous public health programs, authors’ collaboration remained less than 0.5%.

**Fig. 5:**
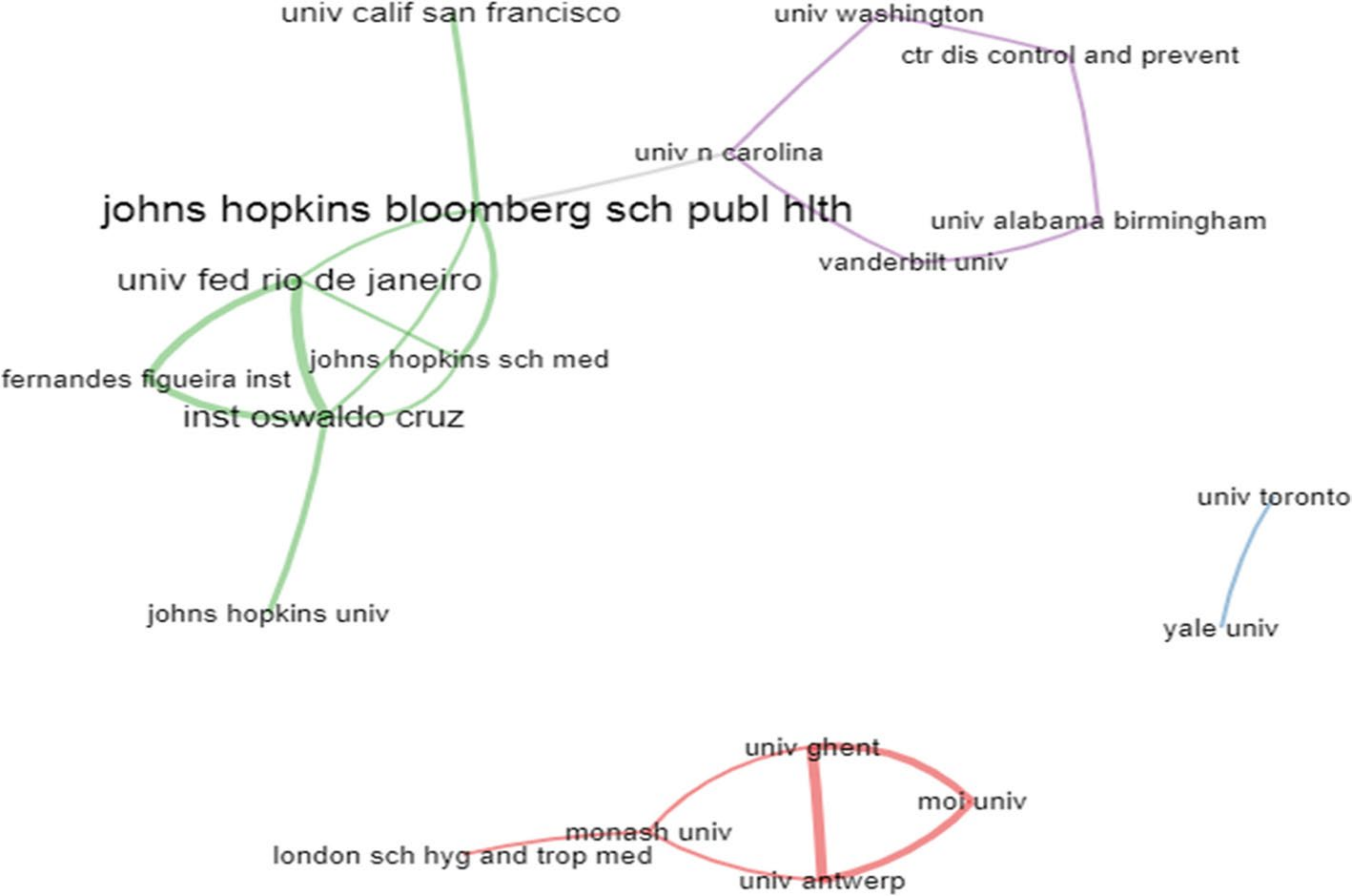
Institutional collaboration network (cluster 1: 5[red], Cluster 2: 2[blue], Cluster 3: 7[green], and Cluster 4: 5[purple]). Source: Bibliosiphiny compilation (set at top 20 collaborations) form Web of Science database

The top 20 institutional collaboration is presented in Fig. 5. The result showed four (4) collaboration clusters with two independent clusters. The largest collaborator was John Hopkins Bloomberg School of Public Health with strong intra- and inter-institutional collaboration. The John Hopkin’s Institution is also linked to cluster 4 (with 5 institutions), which have a maximum of two collaborations within their institution except the University of North Caroline which has three (3) collaborations. The cluster 3 have a better collaboration link among the institutions, and this accounted for the observed thick lines drawn from schools. Intra- and inter- institutional collaboration is seen only in John Hopkins Institutions, with isolated smaller collaborations. Such collaborative network is poor for a clinically related condition of great public health and clinical significance which the scourge of HPV/HIV has exposed the global population.

**Fig. 6:**
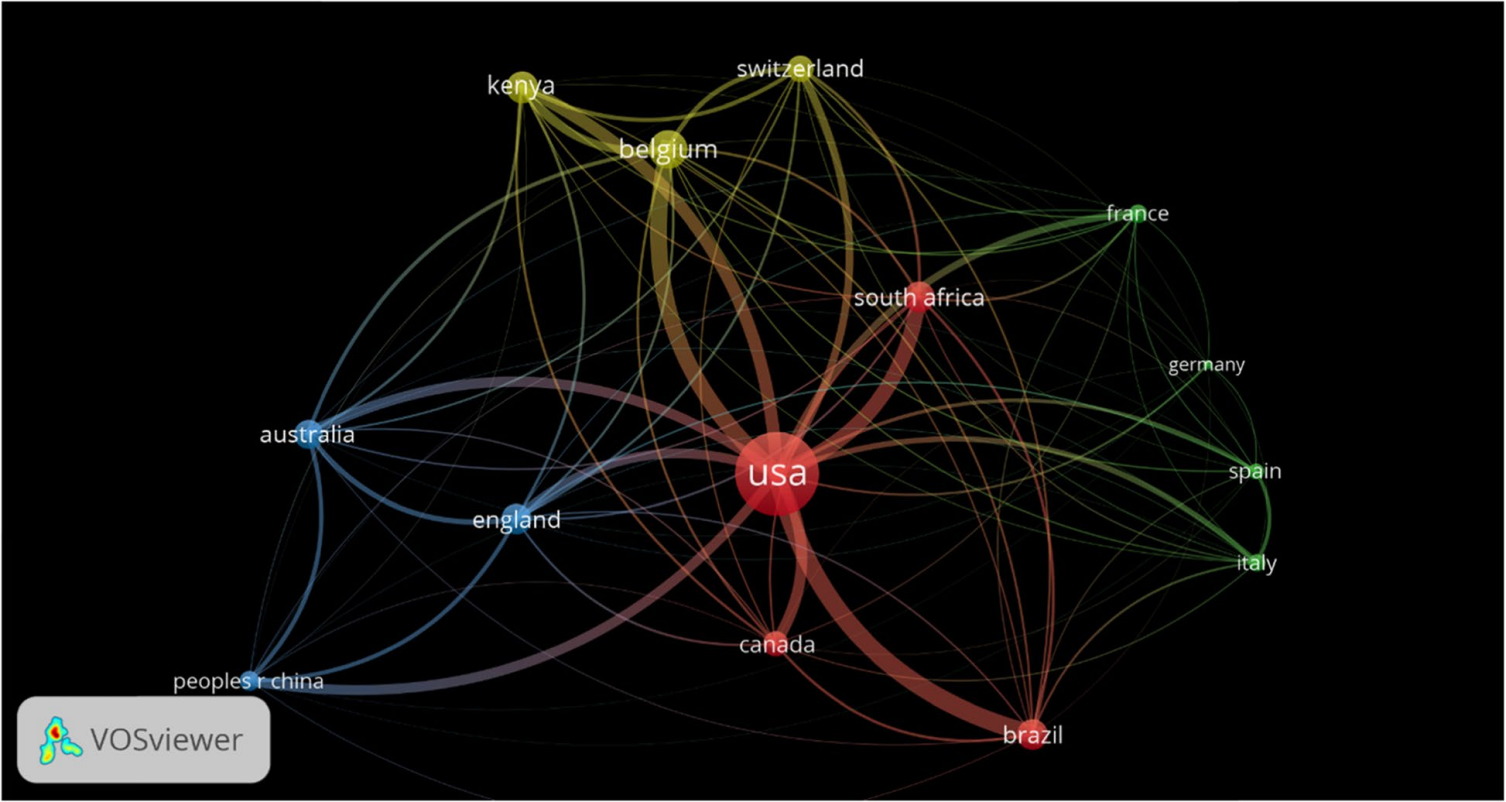
Country collaboration network (cluster 1: 4 [red], Cluster 2: 4[green], Cluster 3: 3[blue], and Cluster 4: 3[lemon],). Source: VOSviewer compilation form Web of Science database. Note: Maximum number of countries per document and minimum number of documents is set at 25 and 7 respectively. Of the 568 countries represented, 14 were met or exceeded the threshold. The bubble size indicates the biggest collaborating country, while the colour represents the clusters and line thickness is the total strength of the corresponding link

In Fig. 6, USA had the largest country collaborating networks, revealing its collaboration activities with all other 13 countries, however, higher degree of collaboration was observed with Brazil and Belgium. Kenya and South Africa also had high degree and/or strong collaboration network with USA as well as Australia, Canada, Switzerland, China, France and England. From the overlay (represented by years of collaboration; masked by total collaborating links), it is noted that interest in collaboration and collaborative studies only arises within 2010 and 2016, with Belgium and Brazil receiving earlier collaborations, while Kenya and South Africa were within 2014 to 2016.

## Discussions

The goal of this study was to provide an overview of global research publications and progress on HIV/HPV coinfection. Thereby identifying authors production/production trends, inter institutional/international research activities, conceptual topic thematic and gaps for future prospects. The findings denote a moderately increased over time number of publications, contributing countries, and the average number of authors per document. However, the number of publications that made significant contributions to the subject, the countries and the average number of citations per document was limited to the developed world.

Furthermore, based on the fact that HPV infection alone is an indication of candidacy for carcinogenesis a chronic immune humiliating disease. When HPV was found in the same individuals with HIV the master of immunodeficiency in humans, then we saw the justification for the magnitude of observed co-infections in several diseases ranging from common benign warts to invasive carcinoma at various anatomical sites of infection, including the cervix, vulva, vagina, penis, anus, and oropharynx.

In response to increasing global epidemic of HIV, limited studies have been gar towards its associated coinfections such as HPV. Globally, approximately 80% of the sexually active women become infected with one of the 13 high-risk HPV (hr-HPV) in their life time [14]. Also, HIV infected women have been found to be three times more likely to have a new HPV infection compared to the non-HIV-infected women [119]. HIV infection is identified as a substantial risk factor for HR-HPV infection [63, 73]. This could be related to immunosuppression creating lower HPV clearance rates, which could impact high rates of HPV infection, persistent HPV infection, or latent HPV reactivation [72]. At the same time, the reactivation risk may increase around age 50 [106]. HPV/HIV co-infection is a primary public health burden in the sub-Saharan African countries resulting to several cervical abnormalities amongst women [22]. However, the latter is often persistent among HIV-infected women, leading to adverse incidence of high-grade squamous intraepithelial lesion (HSIL) [73]. The role of African researchers is paramount to the advances in HPV/HIV co-infection cases especially as it is predominant amongst Africa women which share the huge burden of its prevalence. In bibliometric studies the quantity of scientific publication, represents productivity on a research subject [118]. This implies that changes in the number of published documents as well as reports on a subject, indicates knowledge-scape and/or change/shift in knowledge which also reveals potential strides in control/usefulness of a subject.

However, the progress of studies to curbing the prevalence of HIV/HPV co-infection among women [68] around the world, with greater pecentage in sub-Sahara Africa [60, 91] is poorly represented by the amount of study from the region and is of utmost importance. It is also evident that although the annual scientific publications between 2005 and 2007 were relatively high for HPV/HIV co-infections, the subsequent years recorded more scientific publications indicating that as the years goes by, interest in related studies and research continues to grow within the scientific discipline. Following the reports from Kenya, it may be deduced that although there is increase in interest, the authors in Africa and publication impact remains imprecise as previously reported by various investigators [29, 37, 57, 79].

From the forgoing in Table 4 above, it may be adjudging that publication time line in most cases may not be use in justifying how relevant a study or how experience an author may be considering the 17-year timeline of Tristao A (2002), Williamson AL [134], and Fernandes ATG (2002). One may be quick to say that although the greatest impact of the HPV/HIV co-infection reports were observed in Africa, most of its related studies were reported by researchers and authors from other continents of the globe hence more global citations than local citations. The African researchers and authors of viral studies need to revamp their interest in indigenous research or African related interest with a view to addressing the indigenous science based and public health concerns.

**Fig. 7:**
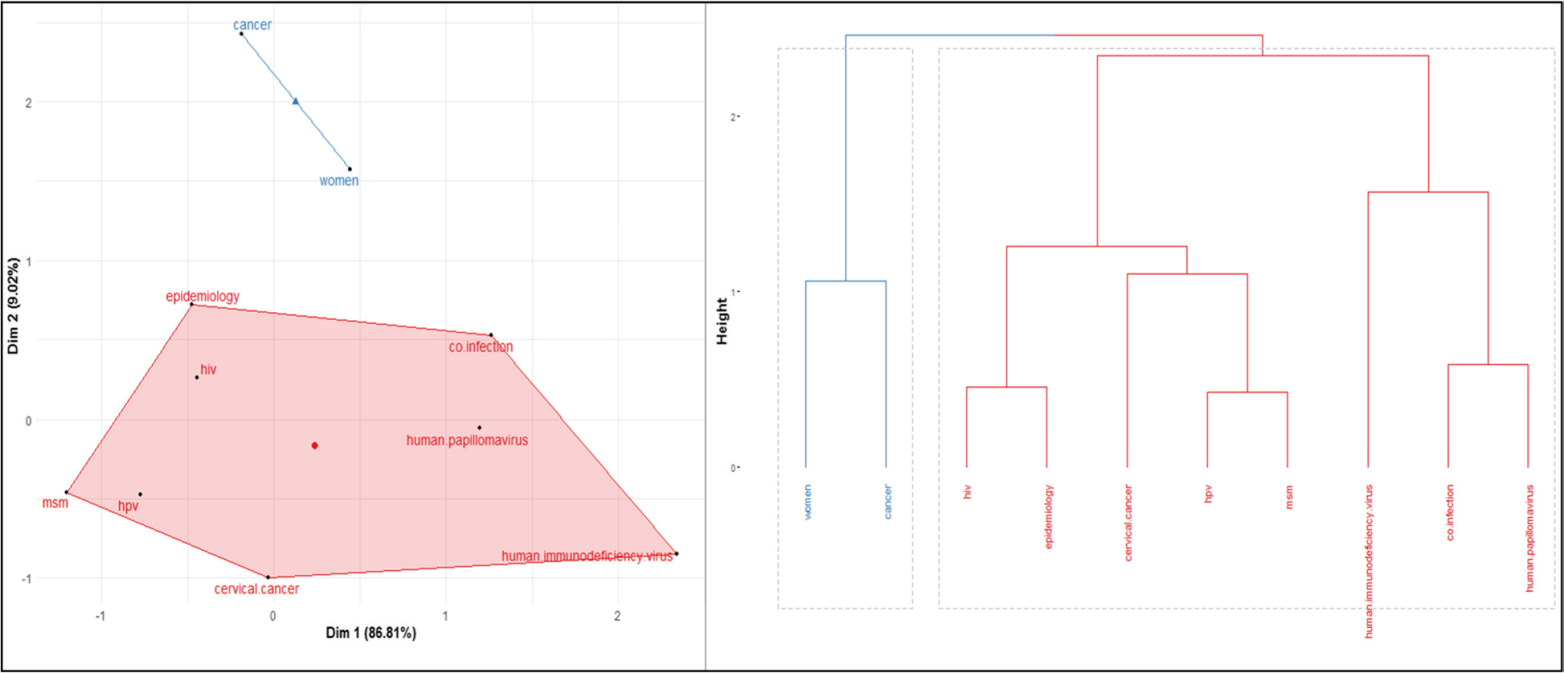
Conceptual Structure map using Multiple Correspondence Analysis and Dendrogram representation of key terminology in the conceptual structure of the relationship between the keywords in HIV/HPV co-infection research is presented in Fig. 7

**Fig. 8:**
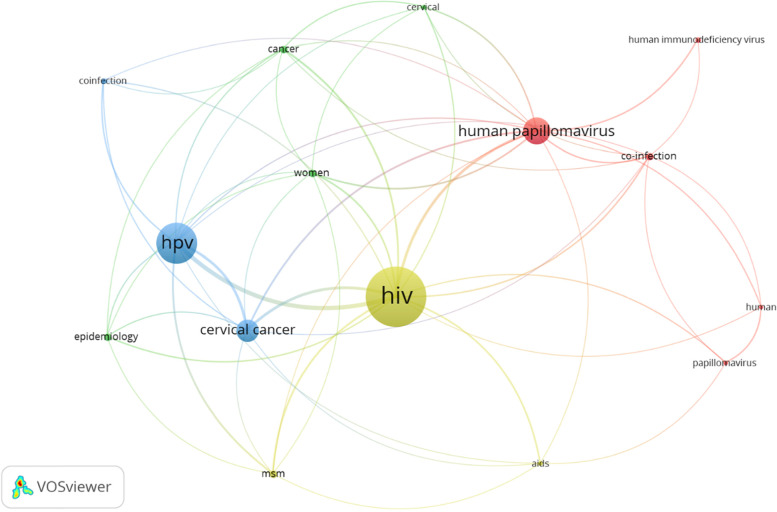
Authors keywords co-occurrence network Cluster 1 (5), [red]; Cluster 2 (4) [green], Cluster 3 (3) [blue], Cluster 4 (3) [lemon]. Note: The minimum number of co-occurrence was set at 5 and of the 428 keywords, 15 met the criteria. The bubble size indicates the most occurring keyword, while the colour refers to the clustering and line thickness the strength of the corresponding link

Here, as expected, the keyword analysis shows the search terms of interest “HIV/HPV/Co-infection” as appeared in the author keywords network (Figs. 7, 8). The most occurring keyword is HIV and this is coupled to HPV. Co-infection was observed to be found in links between HIV and HPV. The mapping analysis identified keywords with a high citation torrent that can indicate research topics gaining much interest. The keyword co-occurrences in our bibliographic meta-synthesis communicate the conceptual co-word structure and discover groups of articles that communicate shared concepts. Nevertheless, the authors keywords co-occurrence network mapping suggests thematic including; cancer in women, epidemiological characteristics research and HPV genotypes thematic implicated in the development of high-grade intraepithelial lesions and invasive carcinoma. In general population, the prevalence of HPV related cervical cancer [38] is high among women living with HIV. AIDS is a defining condition in cervical cancer related to HPV [18]. Also, report has shown [39] an increasing incidence of HPV in HIV patients in men who are involve in sex with men (MSM), as compared to HIV-negative controls. Cluster 1 (5), [red] reveal the risk of HPV-associated co-infection with HIV-infected individuals [54]. Furthermore, understanding of molecular epidemiology of HR-HPV in a given region is needed for vaccination and prevention of genital HPV infections.

Co-citation analysis help to identify documents that contain concept, ideas, experiments, or methods that has a noble recognition, as indicated by their co-occurrence of citations [61]. Hence, knowledge of how documents are cited together aid researchers and practitioners to comprehend the importance of previous contributions that were made within a field study.

In the present day, research collaboration has increased among researchers in different field of studies [25, 26]. Collaborations of either authors, institutions/organizations, countries and within authors have been shown to be high in this study hence, it has given room for knowledge and skills transfer, cutting boundaries, mitigating costs, enhancing greater benefits of research findings, and measurement attributes as previously reported by various investigators [52, 58, 113]. Furthermore, collaboration enhances establishment of research communities that constitute social networks amongst countries (develop, developing or high, middle to low income) and researchers in both resource/resource limited locality. Social networks avail researchers with the tendency to create and share knowledge [13, 31], as well as identify the knowledge domain within disciplines [136]. The collaboration networks analysis was also used to identify key performing authors or institutions or countries to validate the relationships between indicators of these networks and outputs in HIV/HPV co-infections. This also indicates that although reports on HPV/HIV co-infection started in 1992 and sustains till 2019 with repeated increase and rising impact on the African environments, collaborative studies with Africa researchers only started about 6 years ago. This may probably be one of the reasons behind the low Africa output or publications in studies on HPV/HIV co-infection. It may also be another aspect were African researchers’ needs to revamp research-based interest.

### Future Prospect

From the foregoing, it is clear that the African related interest in HPV/HIV co-infection studies is relatively poor, which may have accounted for the low knowledge-scape and progress despite the introduction of vaccination and other diagnostic strides. The authors’ research collaboration network, countries collaboration, authors collaboration, citations and implementation of research-based findings in previous studies are yet to receive the aftermath, especially as various countries in the African continent have not received any related collaboration yet she is reporting co-infection associated with HIV/HPV. African needs to awake and stir up the responsibility of research-based interest in HPV/HIV co-infection studies to resolve indigenous public health concerns.

Nevertheless, an increased attention and interest-based studies on the endemicity and prevalence of HPV/HIV co-infection as well as other viral pathogens be added with a view to addressing the indigenous public health concerns associated with the viral endemicity.

### Limitations

The current study examines HIV/HPV co-infections from a bibliometric perspective. However, there was a sluggish annual growth rate, and the most relevant papers to the subject were written between 2005 and 2019, as measured by the association between the mean/TC per article and the mean total citation (TC) per year. The most prominent authors by research impact were JM Nicol AF and Williamson AL. Plos One and BMC infect. Dis. been the leading journal publishing on HIV/HPV co-infection. The conceptual factorial analysis identified two sub-fields, including cancer in women and HIV/HPV co-infection epidemiology characteristics for research engagement. In addition, the multiple-country publications and cooperation index revealed a substantial network of collaboration among researchers in developing nations. We observed that the world’s most powerful governments are investing in HIV/HPV co-infection research through disbursing funds and increasing mentorship among scholars in the region, which is missing in Africa. This study’s findings may be valuable in identifying potential partners for younger academics interested in joining the field. The study’s strength is evidenced as the first holistic content analysis and systematic science mapping of HIV/HPV co-infection research as a possible tool for widening knowledge with the ultimate goal of lowering the associated viral infection, a public health burden in Africa.

However, the use of a single database may limit the identification of additional pieces of literature. Also, the keywords for data collection may have restricted the scope of the research directions. Using a threshold for co-citation analysis based on the number of total citations may hinder the use of the complete published article within the timeframe.

## Conclusion

In this bibliometric analysis, the results revealed low global research in HIV/HPV co-infection, and the publications of 2005 and 2007 were of more significant scientific relevant research outputs. The network analysis suggests that high-income countries have better interrelationship drives than low- and middle-income countries with a high prevalence of HIV/HPV co-infection and limited collaboration networks.

With the increasing prevalence of HPV and high-risk HPV genotypes among HIV-infected men and women couple with asymptomatic genital distress of different degrees of severities, we recommend a reviewed narrative for future study to elaborate emerging themes and gar research focus.

Countries/Regions with a high incidence of HIV/HPV co-infections requires a long-term follow up of individuals with dysplastic lesions associated with HIV infections.

Based on the analysis, we recommend further scientometrics studies using a combination database involving the mapping of knowledge structure. In contract, future research should be directed towards developing and implementing vaccination and programs to reinforce the current trends of HIV/HPV coinfection. At the same time stakeholders and governments in Africa region to engage in funding and its effective ultiazation for research to the benefit of the African population.

## Data Availability

The datasets used for this study are available from the corresponding author on reasonable request.

## Abbreviations

HIV/HPV: Human immunodeficiency virus / Human papillomavirus
PRISMA: Preferred Reporting Items for Systematic Reviews and Meta-Analyses
TC: Total citation
WHO: World Health Organization
IARC: International Agency for Research on Cancer
AIDS: Acquired immunodeficiency syndrome
ART: Antiretroviral therapy
HAART: Highly active antiretroviral therapy
CIN: Cervical intraepithelial neoplasia
AIN: Anal intraepithelial neoplasia
WoS: Web of Science
